# Developing targets for public health initiatives to improve palliative care

**DOI:** 10.1186/1471-2458-10-222

**Published:** 2010-04-29

**Authors:** Nils Schneider, Sara L Lueckmann, Franziska Kuehne, Katharina Klindtworth, Mareike Behmann

**Affiliations:** 1 Hannover Medical School, Centre for Public Health and Healthcare, Institute for Epidemiology, Social Medicine and Health Systems Research, Carl-Neuberg-Str.1, 30625 Hannover, Germany

## Abstract

**Background:**

Palliative Care is an approach that improves quality of life for patients and their families facing the problems associated with incurable life-threatening illness. In many countries, due to the rapidly ageing population, increasingly more people are suffering from serious chronic disease towards the end of life, making further development in palliative care a major public health challenge. The aim of this study was to develop the first targets for public health initiatives to improve palliative care in Germany.

**Methods:**

Based on the findings from pilot studies (qualitative interviews and surveys with different stakeholders in the health care system), we conducted a modified Delphi study with two rounds of questionnaires with experts in public health and palliative care. In the first round, the experts commented on the findings from the pilot studies. The answers were evaluated descriptively and with qualitative content analysis, resulting in the formulation of 25 targets. These were presented to the experts in the second Delphi round to assess each of them separately with regard to its importance and current implementation (7-point answer scales) and in relation to the other targets (defining the five most important of the 25 targets).

**Results:**

Six most relevant targets for public health initiatives to improve palliative care in Germany were worked out: Supporting palliative care as a basic attitude for the care of people in the last phase of life; coordinating healthcare for people in the last phase of life; establishing cooperation among health professions and disciplines; establishing education in palliative care for all professional groups with contact to people in the last phase of life; reviewing the evidence of palliative care measures; offering support to family members who are caring for someone in the last phase of life.

**Conclusions:**

To systematically develop palliative care, it makes sense to define fields of action with individual targets. For Germany, it can be recommended to give priority to the targets that were highlighted as the most relevant in this study. The next step will be to develop, implement and evaluate tangible measures to achieve these targets.

## Background

Palliative Care is an approach that improves quality of life for patients and their families facing the problems associated with incurable life-threatening illness. The relief of suffering through early identification, assessment and treatment of pain and other physical, psychosocial and spiritual problems is the main goal of palliative care [1]. Although palliative care is most often discussed in the context of end of life care for cancer patients, it is actually appropriate to apply it also at earlier stages of incurable illness, both malignant and non-malignant [2,3].

It is a major undertaking for health systems worldwide to delive appropriate palliative care. However, there are serious deficits in this field in many countries, and the need for palliative care will further increase as a result of demographic development with increasing numbers of older people with incurable chronic disease and multimorbidity. Further development in palliative care can therefore be seen as a public health priority [4,5].

Awareness and recognition of the value of palliative care has become a pressing issue together with political commitment to developing comprehensive palliative care services as an integral part of mainstream health and social care systems, and a better and systematic knowledge base for planning and delivering effective palliative care services [4]. Appropriate policies should take into account the context of culture, disease demographics, socioeconomics, and the specific framework of the healthcare system within each country [6].

In Germany, in recent years scientific policy advice concerning the status of palliative care and potential improvement measures nearly exclusively reflects the perspectives of experts from specialized palliative care and hospice organizations [7], and the political actions towards palliative care have tended to focus on specialist palliative care. An example of this is the establishment of specialist outpatient palliative care (*spezialisierte ambulante Palliativversorgung, SAPV*) as part of the 2007 German health care reforms (§37b of Social Security Code V) without strengthening generalist palliative care at the same time [8]. However, most palliative care occurs in a generalist rather than a specialist palliative care setting involving family, family doctors, community nurses and nursing homes, for example [9,10]. To further develop palliative care as a whole field, integrating specialist and generalist care as well as different levels of responsibilities within the health system, it is important to involve all relevant stakeholders in the policy making process.

Our aim therefore has been to systematically develop targets for public health actions to improve palliative care in Germany on a broad societal and political base, and to include a wide range of stakeholders from different fields.

### Study framework

This article presents the findings of the final stage of a three-step study. In steps 1 and 2, the perspectives of public health and palliative care experts, and the perspectives of different stakeholders in the health care system were analyzed in relation to the problems surrounding palliative care in Germany and potential measures to improve it. In the third step, based on these findings, public health targets for palliative care were developed systematically with experts from public health and palliative care using a Delphi study approach.

The results from steps 1 and 2 have been published elsewhere [11-13] and are summarized as to their relevance to the present work:

Firstly, to gather information on selected palliative care issues, with the focus on Germany, qualitative guided interviews were performed with internationally experienced public health and palliative care experts. Using qualitative content analysis, older people and non-cancer patients were identified as target groups with a particular priority for palliative care. Significant barriers to the further establishment of palliative care were seen, amongst other things, in the powerful lobby groups and the federalism of the German health system [13].

Secondly, to study the views of policy-makers and stakeholders from at the meso and macro level of the German health care system, 442 representatives of organizations and institutions were included (e.g. medical associations, political boards, health insurances). Using a standardized questionnaire, the main topics included the most recent health care reform, quality in palliative care and living will. Most of the respondents (69.9%) rated the recently introduced specialist outpatient palliative care positively. The majority of the interviewees agreed that the effectiveness and efficiency of palliative care services need to be further evaluated. Two thirds believed that political regulations of living wills could help minimize uncertainties concerning end-of-life decisions; palliative care specialists were less likely to be of that opinion compared to the other groups [11]. Furthermore, the participants were asked to evaluate 18 pre-selected improvement measures with regard to their general meaningfulness and the feasibility of their potential introduction into the German health care system. All the improvement measures were rated significantly higher in respect of their meaningfulness than of their feasibility. In detail, the meaningfulness of 16 measures was evaluated positively (70-100% participants chose the answer "good"); for six of these measures feasibility was evaluated negatively (0-30% "good"), while for the remaining ten measures feasibility was evaluated inconsistently (31-69% "good"). The reason why potentially meaningful improvement measures are considered to be not very feasible in Germany may be the existence of barriers resulting from the high degree of fragmentation of health care provision and responsibilities [12].

## Methods

We conducted a modified Delphi study with national experts in public health and palliative care. The Delphi method was chosen as it is very suitable for areas where only uncertain or incomplete knowledge exists, as it allows experts to evaluate the topic in a flexible, iterative multistage group process [14,15]. Originally the Delphi method consists of three rounds of questionnaires. We modified it to two rounds as in the project planning we presumed that the study aims can be achieved in two rounds and that thereby the experts' attendance for participation would be increased. However, we included the option of a third round of questionnaires if the study aims were not sufficiently achieved after two rounds.

### Sampling

We purposively recruited leading German scientists to reflect a range of scientific expertise in the areas of public health and palliative care. The selection of potential participants was based on our own knowledge within the field and on interdisciplinary discussions with other scientists in our institution.

For palliative care, all available academic faculties of palliative medicine in Germany were included (five chairholders) and further experts with nursing and theological background who are representatives of their profession in the German Association of Palliative Medicine. For public health the field is much more difficult to define due to the strong heterogeneity. After intensive discussions we invited chairholders and professors to reflect a range of different professional backgrounds (medicine, economics, nursing, social science) and of different academic organizations (e.g. universities, scientific associations). Our aim was to invite public health experts who have a main area of research in palliative care. However, in practice the research fields are multi-faced as is characteristic for public health.

In total nineteen scientists were asked to participate, of whom sixteen agreed to take part (nine from a specialist background of public health, seven from specialist palliative care). The experts were asked to nominate another person (e.g. research fellow with research focus on end-of-life care) within their institution if they were not able to participate themselves. The participants' demographic data is shown in Table 1.

**Table 1:** Demographic data of the Delphi study participants

	Public health experts*	Palliative care experts*	Both groups
**Participants (N =)**	**9**	**7**	**16**

**Mean age (years)**	52	49	51

**Female**	3	1	4

**Male**	6	6	12

**Current position (N =)**			
Chair holders	7	4	11
Associate Professors	0	1	1
Research fellows	2	2	4

**Primary professional background (N =)**			
Medicine	4	5	9
Nursing	1	1	2
Theology	0	1	1
Economics	1	0	1
Social Science	3	0	3

*with regard to the participants' current position

### First Delphi round

#### Instrument

For the first Delphi round, a semi-structured questionnaire was developed based on the main findings of the pilot studies [11-13]. The pilot studies' findings were repeatedly discussed by the multidisciplinary study team to reach consensus concerning the core topics and the items in the questionnaire.

The questionnaire was self-completed by the experts who were asked to comment on the items in free text answers. Additionally, closed questions were used to assess specific statements (three-point scale: "agree", "disagree", "don't' know" or five-point scale: "very high to "very low"). The instrument was tested with scientists within our institution for comprehensibility and handling. For the final version please see Additional file 1.

#### Analysis

Qualitative data were analyzed with a descriptive approach. Qualitative description is probably the least theoretical of the qualitative approaches. Whereas other qualitative approaches often aim to develop theories and analyze data in a reflective interplay with existing theories, in qualitative description researchers stay relatively close to the data. Qualitative description is founded in existing knowledge, thoughtful linkages to the work of others and clinical experiences [16]. Therefore, this was the most suitable analytic approach in this study.

Three researchers (NSCH, SL, MB) independently read the free text answers to get a first impression of the material. Then two researchers (SL, MB) independently analyzed each free text answer sentence by sentence and coded line by line according to qualitative content analysis [17]. Every code was constantly compared and contrasted as well as grounded in data, and codes were conceptualized into related categories. In the study group there was an ongoing discussion of emerging themes and categories and divergence of interpretation to aim at consensus. There was no set number of priorities before the beginning of the Delphi study.

Quantitative data were recorded with MS Excel (2003) and transferred to SPSS for Windows 16.0 for descriptive analysis. The results were used in the interpretation of the qualitative findings.

The product of the first round was six major categories with 25 subcategories which were formulated in terms of targets for public health initiatives to improve palliative care. Exemplary quotes are given to illustrate a range of opinions of both groups, experts from palliative care and experts from public health. (Additional file 2)

### Second Delphi round

#### Instrument

In the second Delphi round the 25 targets derived form the first round were presented to the same participants. Using a semi-structured questionnaire the experts were asked to assess each of the targets with regard to: (A) its importance and (B) its current implementation. Therefore a 7-point answer scale was used which ranged from "-3" (= of no importance/very poorly implemented) to "+3" (very important/very well implemented). Furthermore, at the end of the questionnaire, the participants were asked to choose the five most important of the 25 targets. The experts were encouraged to explain their assessment in free text form.

#### Analysis

The quantitative data were recorded with MS Excel (2003) and transferred to SPSS for Windows 16.0 for descriptive analysis. The medians of judged importance and current implementation were calculated for each target. Furthermore, the absolute frequency of choices of the five most important targets was determined. Spearman's rank correlation coefficient was calculated to establish the relationships between level of importance and the number of mentions in the choices named as the five most important targets.

Free text answers were used to better understand and conceptualize the quantitative data without specific qualitative analysis.

#### Ethics

The study was conducted with the approval of the ethics committee of Hannover Medical School (letter dated 26 February, 2007).

## Results

### First Delphi round

In the first Delphi round 25 targets for public health initiatives to improve palliative care in Germany were developed and structured into six subject areas (major categories). Additional file 2 presents the targets together with exemplary quotes to illustrate a range of opinions of both groups, experts from palliative care and experts from public health. Additional file 3 shows the descriptive assessment of the closed items. In the following the results are summarized on the basis of the major categories.

#### Palliative care approach

There was strong agreement to the palliative care World Health Organization's definition of palliative care. 81.2% agreed that the quality of a health care system is measured against how it delivers care for severely ill and dying people. However, it also became clear that it is necessary to sharpen the concepts in palliative care and to clarify differences as well as overlaps with other areas of the health care system. From the expert statements it was worked out that palliative care should be more than an approach to care but should be a basic attitude.

#### Patient and family

The needs of family members who are caring for someone in the last phase of life were addressed in many ways. For example, it was pointed out that family members need more information about professional home care services. The information needs to be specific for the target group considering the cultural and social background of patients and families to overcome socio-demographic barriers that are well known from other areas of the health system, e.g. prevention. Job-related disadvantages should be reduced if family members lay claim to their legal right to unpaid leave of work to take care of someone. In many statements it was pointed out that the quality of life of the people concerned needs to be the focus of all measures in palliative care.

#### Health services

Differences became obvious among the experts concerning specialist palliative care teams. On the one hand, the value of the teams was emphasized, e.g. the reduction of hospital admissions. On the other hand, there was concern that new specialised services may create new interfaces with problems in coordination and defining responsibilities. Overall, it became clear that both areas specialist and generalist palliative care are in need of improvement in Germany, particularly regarding outpatient services. However, 75% of the participants agreed that good generalist palliative care reduces the need for specialist palliative care.

The experts strongly emphasized the need for better cooperation among health professionals of different disciplines, and for different models of care considering regional structures (urban, rural). The major target group for palliative care service planning was seen in older people due to the demographic changes.

#### Information and qualification

The requirement for information and qualification was worked out for patients and informal carers as already described in the category 'patient and family', and for health professionals. For example, the qualification standards in palliative medicine currently vary significantly between different regions (German federal states), calling for some unification. Health professionals who are not specialised in palliative care but provide care for severely ill and dying people within other settings (e.g. GP's, oncologists, community nurses) should have a basic knowledge in palliative care.

#### Research

Some experts criticised a lack of evidence in palliative care and a lack of appropriate quality indicators. For example, in political debates the number of hospices and palliative care units available in Germany is often used as a measure of the quality of palliative care in the health system. There was a call for more patient centred research considering different target groups beyond cancer.

#### Financing

This category has two dimensions, firstly reducing the financial burden of family members who take care of someone and secondly, overcoming the traditional fragmentation of the service funding in the German health system, e.g. the not matched funding of outpatient and inpatient services.

### Second Delphi round

In the second Delphi round each of the targets was assessed with regard to its importance and its current implementation. The final results are shown in Additional file 4. The major findings are summarized in the following.

#### Importance of individual targets

The median importance of the individual targets ranged between 2 and 3. In total, four targets achieved the highest possible median (3 = very important): 'Supporting palliative care as a basic attitude for the care of people in the last phase of life' (Target 3), 'Centering the quality of life of the people concerned' (Target 8), 'Establishing cooperation among health professionals and disciplines' (Target 9), 'Establishing education and training (Target 19)'.

Asked for the most important targets, seven targets were mentioned most frequently, defined as mentioned by at least one third (N ≥ 5) of the participants: 'Supporting palliative care as a basic attitude' (Target 3), 'Offering support to family members who are caring for someone in the last phase of life' (Target 4), 'Centering the quality of life of the people concerned' (Target 8), 'Establishing cooperation among health professionals and disciplines' (Target 9), 'Coordinating healthcare' (Target 11), 'Establishing education in palliative care for all professional groups with contact to people in the last phase of life' (Target 19), and 'Reviewing the evidence of palliative care measures' (Target 21).

There was a relatively strong link between the median for the evaluation of importance and the number of mentions (r = 0.6); the targets that were assessed as very important were accordingly very often chosen as one of the five most important.

#### Status of implementation

The median for current implementation of the individual targets was between -2 and +1. Only one target was assessed positively regarding the status of their implementation: 'Centering the quality of life of the people concerned' (Target 8, Median 1.0).

## Discussion

The first public health targets for palliative care in Germany have been developed. A strength of this multi-stage project is the participation of a variety of stakeholders from different levels of the healthcare system, including, e.g., representatives of the political sphere, sickness funds, medical and nursing organizations and national and international scientists in the specialist areas of public health and palliative care. The results therefore reflect a broad spectrum of different perspectives which clearly extends beyond the opinion-forming of smaller expert groups. This wide range should be important for acceptance of the targets by the different addressees in society and in the healthcare sector.

The 25 targets were classified in the following six superordinate subject areas: *palliative care approach, patient and family, health services, information and qualification, research, financing*. The content of these subject areas corresponds in large part with Rao et al. [18] who identified nine clusters of end-of-life initiatives in Georgia, USA. In contrast to Rao, in our study the area 'Policy' was not identified as a separate subject area, although it did play a role as cross-sectoral theme in all other areas.

In 2007, the European Association of Palliative Care (EAPC) initiated the Budapest Commitments. These provide a framework including potential commitment goals for the national associations. The framework covers five areas in the development of palliative care: access to medication, policy, education, quality, and research [19]. These areas are addressed by the results of our study, with the exception of 'access to medication'. This is plausible because the availability and accessibility of painkillers and other medication used in palliative care constitute no fundamental problem in Germany.

As a consequence of the Budapest Commitments, the charter process for the care of very severely ill and dying people was initiated in Germany in 2008 [20]. The charter process aims to support both dialogue between all parties involved and the greater public discussion. It also aims to give future developments a direction, and to agree common targets and actions through a consensus process. The results of this study will be incorporated into the charter process and will thereby be directly linked to the current political debate about palliative care.

### Most relevant targets

There were targets that were rated high individually but not frequently mentioned as one of the five most important (e.g. Targets 14 and 22). It seems that these targets are seen to be generally very important, but lose priority when compared to the other targets.

In contrast, seven targets selected by at least one third of the participants as the most important compared to the other targets were also rated with a high to very high importance individually (Targets 3, 4, 8, 9, 11, 19, 21), which means that these targets can be identified as most important, whether looked at separately or in relation to the other targets.

In the experts' opinions, none of these targets are currently very well implemented. However, one target (no 8) was at least slightly positive evaluated with regard to its implementation status.

Therefore, overall, there are six most relevant targets for public health actions to improve palliative care in Germany:

### Supporting palliative care as a basic attitude

Measures to improve palliative care tend to focus on a form of care that in Germany until now is offered mainly in selected institutional settings like specialized hospital units or hospices. This has coincided with claims for more palliative care services. However, a broader angle is needed to achieve sustainable improvements in the care for severely ill and dying people. It will not suffice to establish more specialist services. Changes are rather required in each and every area of health care to achieve a level of successful and needs-based end-of-life care. This includes that we have to encourage societal and scientific debates about dying, death and end-of-life care, which are still largely non-issues [21]. Promoting the concept of palliative care may contribute to overcoming the curative paradigm in medicine in situations where it is more appropriate to accept the process of dying and to offer best supportive palliative care.

### Coordinating health care

The fragmentation of care across different health care sectors (e.g. inpatient and outpatient care, rehabilitation) is well known as a major problem, and shortcomings in coordination of care are among the most serious problems in Germany [22,23]. This problem is not exclusive to palliative care, however, it is particularly evident in this field, as characteristically large numbers of providers and services are involved within a relatively short timeframe (e.g. family doctors, oncologists, palliative medicine specialists, social workers, pharmacies, and nursing teams).

There is no doubt that improvements in coordination and management are necessary. However, the crucial question remains as to what form should the management take? For example, the PhoenixCare intervention shows that home-based case management provided by registered nurse case managers may, in coordination with patients' existing sources of medical care, improve the care of chronically, severely ill patients [24]. A prominent example of a fully integrative model including generalist and specialist palliative care in- and outpatient service, is the Edmonton Regional Palliative Care Program [25]. For example, physician-nurse palliative care consult teams assist family doctors throughout the patient's illness. Since the introduction of the program there was a shift from deaths of cancer patients in acute care hospitals to hospice and home care settings in the Edmonton region which can be taken as an indicator of good, patient-centred quality in palliative care, and the costs of care for these patient groups could be reduced [26].

The experience from other countries shows that, in principal, the fractionation of palliative care can be overcome by new models of care. However, it has to be examined if evidence gathered in one health system can be adapted to another health system as the framework differs significantly e.g. concerning the structure of social security systems and the role perception of patients and health professionals. By all means, the reservations of the stakeholders with regard to new elements of care should be taken seriously. A previous German survey revealed that case management tended to be assessed negatively by many stakeholders, in particular by doctors [12]. This is not surprising as the traditional hierarchical structure of the German health system and its role definitions makes the acceptance of nurses and other non-medical professional groups as case managers difficult to achieve on the part of the doctors. There are many reasons why strategies to optimize coordination should not be decreed politically as top-down policy, but should be developed from care provision considering existing, regionally developed structures.

### Establishing cooperation

A previous study demonstrated that lack of teamwork and conflicting role definitions between doctors and nurses and family practitioners and medical specialists are central problems affecting the provision of appropriate palliative care in Germany. Furthermore, the distribution of financial resources for palliative care within the German health care system is a controversial issue. For example, general practitioners and geriatricians have stated that new institutions and services could be created to exploit a lucrative new area of business in palliative care. It seems that interdisciplinary rivalry and strong lobbyism - well-known in other fields of the German health care system - have increasingly invaded palliative care now that it has become a new specialized area requiring appropriate funding from the statutory health insurance system [27].

It is disheartening but undeniable that the reality in the health care system leaves the high demands of palliative care only partly fulfilled. Hardly any other discipline has such a markedly multidisciplinary approach as palliative care has, seeking to integrate the expertise of doctors, nurses, social and welfare professionals, psychologists, counselors and those providing spiritual support, and to care for both the patients and their family or friends [4]. It is, however, exactly this high demand that encourages the belief that existing obstacles can be overcome, if the concept of palliative care is firmly positioned in the perceptions of all relevant stakeholders.

### Establishing education

Specialist training and basic knowledge and skills in palliative care for all health professionals dealing with people in the last phase of life are required. Despite remarkable advantages in recent years, there is still much room for improvement. For example, in Germany doctors of all clinical disciplines have the opportunity to sub-specialize in palliative medicine, which may only be relevant for a fairly small number of professionals, e.g. aiming at working in specialist services such as palliative care teams or hospices. In contrast, so far palliative medicine has not been a compulsory field in the study of medicine, which leads to most medicine students getting into the job without any noteworthy knowledge of palliative care. This is set to change in the future, however, as the German government recently passed a law in 2009 stipulating compulsory training for medical students in palliative care. It can nonetheless be assumed that it will take many years for this to have a positive impact on medical care.

Awareness and skills training of all relevant health professionals and informal carers are important issues that need to be moved into focus.

### Reviewing the evidence

Up till now research on palliative care has been focused on the provision of care in specialist settings such as hospices, palliative care units and palliative care teams. Furthermore, much of the existing evidence is from pilot projects and academic centres. In contrast, very little is known about the much larger field of primary palliative care delivered by general practitioners and community nurses [9,11]. This dilemma highlights the extensive need for health services research related to the routinely delivered care outside specialist settings and in the community.

### Offering support to family members

Taking care of severely ill and dying patients creates several problems among caring family members. Anxiety and depression are common problems for family caretakers [28]. Families often do not know how to provide physical care and to cope with the losses faced during the illness and dying process and after death. Many caregivers themselves have physical illnesses that impact upon their ability to care for the patient [29]. This problem will become even more pressing in the future with demographic changes leading to increasing numbers of older people with chronic disease and need for care.

A major task from a public health point of view is shaping the societal framework in favour of appropriate working and living conditions that allows families to care for their loved ones - not only at the end of life - and ensuring that the families receive professional support from medical, nursing and social services. The challenge is to make use of the potential within families for the care of ill and dying patients without overwhelming them.

### Limitations

The sample in this study is relatively small. A selection bias is possible as the recruitment was predominantly based on our own knowledge in the field. We tried to reduce bias by intensive iterative discussion within our interdisciplinary study group (NSCH is assistant professor of public health and works clinically in general practice and palliative medicine; SL is economist; FK is psychologist and works clinically in palliative care; KK is health scientist; MB is social scientist). In addition, we sought external advice and supervision from public health and palliative care specialists at Hannover Medical School during recruitment and analysis.

The majority of the participants have a medical background. For the palliative care experts this is due to the fact that in Germany palliative medicine is academically much better developed than other relevant disciplines, e.g. palliative nursing. Only two experts in this study were specialized in nursing which falls short of the significant role of palliative nursing; this perspective may be underrepresented in the findings. However, the professional background of the public health experts was multi-faceted as is characteristic in this field.

Another limitation may be due to a limited understanding of palliative care on the part of some public health experts, and likewise, a limited understanding of public health concepts on the part of some palliative care experts. However, there was strong agreement to the WHO-definition of palliative care among the participants which might indicate the acceptance of the approach and a relatively homogenous basic understanding. Furthermore, it was the aim of the study to develop targets for public health actions to improve palliative care considering the experts' views and opinions from different background and with different priorities.

## Conclusions

Palliative care is a major issue for public health. The importance of the field will further increase with the numbers of older people with incurable, progressive diseases in need of care. To systematically improve palliative care on a public health level, it makes sense to define superordinate fields of action with individual targets. For Germany, it can be recommended to give priority to the targets that were highlighted as the most relevant in this study. Particularly, a stronger focus is needed on the broad field of primary palliative care which, compared to specialist palliative care, is widely disregarded by the general public, politics and research. The next step will be to develop, implement and evaluate tangible measures to achieve these targets.

In addition, there is the need to conduct large-scale surveys of representative samples of stakeholders on perceived opportunities, barriers, and also on structure and availability of different services with international comparisons which take into account the framework of the different health care systems. In this regard the European Association of Palliative Care has conducted some excellent preliminary work, e.g. by initiating the Budapest Commitments that give the opportunities on national and international levels to further develop palliative care in politics, science and practice.

## Competing interests

The authors declare that they have no competing interests.

## Authors' contributions

NSCH conceived the study, recruited the participants, developed the questionnaires, supervised the analyses, interpreted the data and drafted the manuscript. SLL and MB helped to recruit the participants and to develop the questionnaires, performed the analyses and made contributions to the manuscript. FK made contributions to the analyses. KK made contributions to the manuscript. All authors read and approved the final manuscript.

## Pre-publication history

The pre-publication history for this paper can be accessed here:

http://www.biomedcentral.com/1471-2458/10/222/prepub

## Supplementary Material

Additional file 1 **Questionnaire used in the first Delphi round**. The questionnaire which was used for the first Delphi round is shown, translated from German. Click here for file

Additional file 2 **Categories and subcategories developed in the first Delphi round**. The table presents the major categories and the subcategories that were developed in the first Delphi round. Quotes are given to illustrated a range of opinions of both groups palliative care and public health (PH = Public health expert, PC = Palliative care expert). Click here for file

Additional file 3 **Findings of the closed questions in the first Delphi round**. The table shows the descriptive assessment of the closed items in the first Delphi round. Click here for file

Additional file 4 **Targets, importance and implementation**. The table presents the targets for public health initiatives to improve palliative care in Germany, each with its importance and its implementation as assessed in the second Delphi round. Click here for file
