# Supplementary material for: Developing targets for public health initiatives to improve palliative care
Source: BMC Public Health. 2010 Apr 29;10:222. doi: 10.1186/1471-2458-10-222 (PMC2874778; doi:10.1186/1471-2458-10-222)
Supplement: Additional file 1 — Questionnaire used in the first Delphi round. The questionnaire which was used for the first Delphi round is shown, translated from German. [file 1471-2458-10-222-S1.PDF]

Questionnaire for the first Delphi round

1. Please indicate whether you agree with the following statement. “The quality of a healthcare system is measured against how it cares for severely ill and dying people.”

☐ I agree. ☐ I disagree. ☐ I don’t know.

2. The World Health Organisation (WHO) defines palliative care as follows:

> “Palliative care is an approach to improve the quality of life of patients and their families facing the problems associated with life-threatening illness, through the prevention and relief of suffering by means of early identification and impeccable assessment and treatment of pain and other problems, physical, psychosocial and spiritual.”

3. Please indicate whether you agree with this definition of palliative care.

☐ I agree. ☐ I disagree. ☐ I don’t know.

4. In your opinion, how should the definition be changed or what could be added to it?

➔ Answer: ____

Our survey of representatives of associations and institutions of the German healthcare system has shown that the level of agreement with the goals and contents of palliative care (according to the WHO definition) differs. One result is that representatives of funding agencies struggle to see psychosocial care and the involvement of relatives as an integral part of palliative care.

5. Do you think it is necessary to increase the sensitivity of funding agencies for aspects like psychosocial care and assistance for relatives? How could this be done?

➔ Answer: ____

6. 60% of the interviewed representatives of associations and institutions in the German healthcare system held the opinion that the advancement of palliative care is an adequate answer to the demand for euthanasia.

How do you interpret this survey result?

➔ Answer: ____

7. There is an increasing differentiation between general and specialist palliative care. General palliative care describes the care provided by primary structures (e.g. GPs, nursing services, general hospital wards). Specialist care, on the other hand, is provided by structures and people with a specific qualification and a focus on palliative care (e.g. outpatient palliative care services, hospital palliative care units, hospices).

8. The majority of participants in our survey (61%) describe the differentiation between general and specialist palliative care difficult in practice.

How do you interpret this survey result?

➔ Answer: ____

9. Please indicate whether you agree with the following statement.
"Good generalist palliative care reduces the need for specialist palliative care."

☐ I agree. ☐ I disagree. ☐ I don't know.

10. Have you got any comment to this statement?
____

11. Currently there are approx. 332 hospices and hospital palliative care units in Germany with a total of 2,826 beds (= approx. 34 beds/1 million people), 60 outpatient palliative care services and 1500 ambulant hospice services.

What is your opinion on this availability with regard to care tailored to the needs of severely ill and dying people?

➔ Answer: ____

12. The majority of participants in our survey (75 %) held the opinion that the nearby availability of hospice beds was a criterion for good palliative care.

In your opinion, how should "nearby hospice beds" be defined?

➔ Answer:____

13. In your opinion, how far does the criterion of nearby availability apply not only to hospice beds but also to hospital palliative care units and outpatient palliative care services when thinking of good palliative care?

➔ Answer:____

14. Approx. 90 % of patients in German hospices and hospital palliative care units are suffering from a tumour, while a significant minority of patients suffer from non-oncological illnesses.

15. Please indicate whether you agree with the following statements: The small percentage of non-oncological patients in hospices and palliative wards results from

…the fact that it is difficult to predict the course of disease in comparison to oncological patients.

☐ I agree. ☐ I disagree. ☐ I don't know.

… the fact that the need for care of non-oncological patients goes unnoticed.

☐ I agree. ☐ I disagree. ☐ I don't know.

… the fact that hospices and hospital palliative care units favour oncological patients.

☐ I agree. ☐ I disagree. ☐ I don't know.

…the structure of financial support and foundations in the area of palliative care in Germany.

☐ I agree. ☐ I disagree. ☐ I don't know.

16. Have you got any comment to these statements:

➔ Answer:____

17. It is a central challenge for society and in politics to optimise the care for older people suffering from chronic illnesses and polymorbidity. Please indicate whether you agree with the following statement. “The advancement of palliative care plays a central role for the optimisation of the care for older people suffering from chronic illnesses and polymorbidity.”

☐ I agree. ☐ I disagree. ☐ I don’t know.

18. The development of specialist palliative care is less important than improvements in general palliative care to optimise the care for older people suffering from chronic illnesses and polymorbidity.

☐ I agree. ☐ I disagree. ☐ I don’t know.

19. Have you got any comment concerning “palliative care of older people“:

➔ Answer:____

20. The majority of participants in our survey agreed that socially deprived people should be informed systematically about specialist care availability. In which areas do you see chances and obstacles with regard to palliative care for socially deprived people?

➔ Answer:____

21. About half of our interviewees held the opinion that the current offers of palliative care were not sufficiently geared towards the needs of migrants. In which areas do you see opportunities and obstacles with regard to palliative care for people with a migration background?

➔ Answer:____

22. The majority of interviewees (81 %) held the opinion that the current categorisation of the public long term care insurance is not sufficiently geared towards the specifics of palliative patients. How do you interpret this survey result?

➔ Answer:____

23. Please indicate whether you agree with the following statements about the legal entitlement to unpaid leave of absence for a maximum of six months for employees who take care of relatives.

24. This legal entitlement will help to improve domestic care of severely ill and dying people.

☐ I agree. ☐ I don’t agree. ☐ I don’t know.

25. Many employees will not take the unpaid leave for fear of disadvantages in the workplace.

☐ I agree. ☐ I disagree. ☐ I don’t know.

26. Which opportunities and problems do you see in the practical implementation of the entitlement for unpaid leave for the care of relatives?

➔ Answer:____

27. The health care reform of 2007 introduced the entitlement to specialist outpatient palliative care (SAPV). The majority of participants (71 %) in our survey were optimistic that, due to the law, the care of severely ill and dying people would improve. Do you share the optimism? Which opportunities and risks do you see?

➔ Answer:____

28. There are different evaluations with regard to the sense of the advance directive. For example, the majority of representatives of care associations (88 %), funding agencies (86 %) and politics (78 %) held the opinion that the advance directive eliminates the insecurities of doctors and relatives of patients who are unfit to make their own decisions, but only a minority of representatives of hospice and palliative services (44 %) share this view. How do you interpret this result?

➔ Answer:____

29. It is currently being intensely debated which quality requirements should be applied to palliative care. In your opinion, how high should the quality requirements be in the following areas?

Award of the qualification in palliative medicine for doctors

☐ very high ☐ high ☐ medium ☐ low ☐ very low

Award of the qualification palliative care for caretakers

☐ very high ☐ high ☐ medium ☐ low ☐ very low

Specialist outpatient palliative care teams (SAPV according to the health care reform 2007)

☐ very high ☐ high ☐ medium ☐ low ☐ very low

Have you got any comment concerning the quality requirements of palliative care?

➔ Answer:____

30. In your opinion, which are the most urgent research questions in the area of palliative care?

➔ Answer:____

31. In your opinion, which disciplines should be encouraged to pursue research into palliative care?

➔ Answer:____

32. Have you got more comments on this first Delphi-round or suggestions for the following round?

➔ Answer:____
