# Supplementary material for: Developing targets for public health initiatives to improve palliative care
Source: BMC Public Health. 2010 Apr 29;10:222. doi: 10.1186/1471-2458-10-222 (PMC2874778; doi:10.1186/1471-2458-10-222)
Supplement: Additional file 2 — Categories and subcategories developed in the first Delphi round. The table presents the major categories and the subcategories that were developed in the first Delphi round. Quotes are given to illustrated a range of opinions of both groups palliative care and public health (PH = Public health expert, PC = Palliative care expert). [file 1471-2458-10-222-S2.DOC]

| **Major categories** | **Subcategories (targets)** | **Quotes (examples)** |
| --- | --- | --- |
| **palliative care approach** | Sharpening definitions and concepts in palliative care | - *I don’t agree with the WHO definition, because the services that are listed should be available to EVERYONE who needs them. People not suffering from a life-threatening illness are entitled to all mentioned services. I think it is quite cynical to offer the services only in cases where the lifetime is drastically limited.* [PH-12] - *The differentiation between generalist and specialist palliative care is only a little helpful from the perspective of care and healthcare science.* [PH-6] - *Palliative medicine is more than an approach to care. It is an attitude that can develop answers to urgent questions in an increasingly older society with limited resources.* [PC-14]      - *Our own surveys amongst patients with brain tumours showed that many relatives were not sufficiently informed about the available support for care at home. This could be the same for relatives of other patient groups.* [PC-15] - *Problem: insufficient protection of the entitlement to an equal workplace without disadvantages for professional development; a stricter legal enforcement could lead to serious economic problems particularly in small and medium-sized businesses.* [PH-10] - *There is the danger that known barriers of status and social stratum will also apply in the areas of prevention and when claiming certain services. Therefore disadvantaged people should be informed not only via brochures, posters etc but also through means geared towards their environment, milieu. Cultural specifics are also important. (…) Closely linked to this I see the opportunity to learn from other cultures and their ways in dealing with pain, death and dying. It is for example known that social support is readily available.* [PH-11] - *Specialised services create new interfaces, which often create problems.* [PH-12] - *Ambulant specialist palliative care can support patients, their families as well as GPs and care services given a good network and integration. Patients can be taken care of with ambulant services for as long as possible and unnecessary hospital stays and confinements can be avoided and it is possible to die at home if wanted.* [PC-14] - *Different models of care are imaginable. For example in areas like Mecklenburg-Vorpommern or Brandenburg care via community nurses.* [PH-5] - *Regional specifics need to be considered in order to be able to respond to care that is tailored to specific needs.* [PC-14] - *But there are definitely regional over- and undersupplies.* [PC-18] - *The area of ambulant palliative care should be developed further particularly to offer more people the opportunity to obtain care in their domestic surrounding.* [PH-11] - *I see the bigger deficits in the area of general palliative care that looks after the majority of patients and will do so in future.* [PH-5] - *The further development of palliative care has so far not included older patients with chronic illnesses and polymorbidity.* [PC-19] - *Demographic developments determine that in future general palliative care has to be strengthened specifically. This includes education and training as well as the financing of general palliative care.* [PC-14] - *Information has a high value (…) but one should not adopt a naïve information policy, as socially deprived and underprivileged sections of the population are not regarded “hard to get through to” without reason.* [PH-6] - *Of course, a minimum of quality in training and education has to be provided, but I would not set the standards too high. First and foremost it is important that more doctors and caretakers have a basic knowledge in this area.* [PH-5] - *To offer sufficient, high quality palliative care, it is necessary to attract highly qualified employees.* [PC-14] - *I know from my own experience that the requirements for quality are currently very different between the federal states. Therefore a consistent standard should be determined first.* [PC-15] - *I would not want to use the number of hospices and palliative wards as an indicator for care that is tailored to specific needs of critically ill and dying people. It has (...) to be clarified whether the people subject to such specialist services really benefit and want such a form of care.* [PH-12] - *On principle there is the issue of assessment criteria and the relation between general and specialist palliative care.* [PH-6] - *There is a tradition that at first palliative care focussed very strongly on oncological patients and the first support programmes were geared towards this group of patients as well.* [PC-20] - *Many will not be able to financially afford to stay at home for 6 months on unpaid leave to take care of a relative. There has to be compensation (at least partially). This cannot be* *provided by the nursing allowance.* [PC-17] - *The fragmentation of financing has to be overcome* [PC-16] |
|  | Clarifying differentiations and overlaps of palliative care with other disciplines |  |
|  | Supporting palliative care as a basic attitude for the care of people in the last phase of life |  |
| **patient and family** | Offering support to family members who are caring for someone in the last phase of life |  |
|  | Reducing job-related disadvantages of family members who take care for someone in the last phase of life |  |
|  | Considering the cultural background of people is the last phase of life |  |
|  | Considering the financial situation of people in the last phase of life |  |
|  | Prioritising the quality of life of the people concerned |  |
| **health services** | Establishing cooperation among health professions and disciplines |  |
|  | Linking structures of generalist and specialist palliative care services |  |
|  | Coordinating healthcare for people in the last phase of life |  |
|  | Deploying community nurses in the care for people in the last phase of life |  |
|  | Considering regional differences in the determination of requirements for care structures |  |
|  | Planning the demand for specialist palliative care services |  |
|  | Focusing on outpatient palliative care services |  |
|  | Strenghtening primary palliative care |  |
|  | Focusing on the needs of older people in primary palliative care |  |
| **Information and qualification** | Informing about contents, targets and opportunities of palliative care on a societal level |  |
|  | Establishing education in palliative care for all professional groups with contact to people in the last phase of life |  |
|  | Standardize qualifications of specialist palliative care professionals |  |
| **Research** | Reviewing the evidence of palliative care measures |  |
|  | Conducting demand research with regard to specialist palliative care services |  |
|  | Specifying target groups and their need in palliative care |  |
| **Financing** | Reducing the financial burden for family members whotake care of people in the last phase of life |  |
|  | Overcoming structural fragmentation of the funding of services in the health system |  |
