# Supplementary material for: Developing targets for public health initiatives to improve palliative care
Source: BMC Public Health. 2010 Apr 29;10:222. doi: 10.1186/1471-2458-10-222 (PMC2874778; doi:10.1186/1471-2458-10-222)
Supplement: Additional file 3 — Findings of the closed questions in the first Delphi round. The table shows the descriptive assessment of the closed items in the first Delphi round. [file 1471-2458-10-222-S3.DOC]

| **Item** | **Agree** | **Disagree** | **Don`t know** |
| --- | --- | --- | --- |
| “The quality of a healthcare system is measured against how it cares for severely ill and dying people.” | 81.2% (n=13) | 6.2% (n=1) | 12.5% (n=2) |
| “Palliative care is an approach to improve the quality of life of patients and their families facing the problems associated with life-threatening illness, through the prevention and relief of suffering by means of early identification and impeccable assessment and treatment of pain and other problems, physical, psychosocial and spiritual.” | 87.5% (n=14) | 12.5% (n=2) |  |
| “Good generalist palliative care reduces the need for specialist palliative care.” | 75.0% (n=12) | 12.5% (n=2) | 12.5% (n=2) |
| “The small percentage of non-oncological patients in hospices and palliative wards results from … | | | |
| … the fact that it is difficult to predict the course of disease in comparison to oncological patients.” | 25.0% (n=4) | 56.2% (n=9) | 18.8% (n=3) |
| … the fact that the need for care of non-oncological patients goes unnoticed.” | 93.8% (n=15) | 6.2% (n=1) |  |
| … the fact that hospices and hospital palliative care units favour oncological patients.” | 31.2% (n=5) | 31.2% (n=5) | 37.5% (n=6) |
| …the structure of financial support and foundations in the area of palliative care in Germany.” | 62.5% (n=10) | 12.5% (n=2) | 25.0% (n=4) |
| “The advancement of palliative care plays a central role for the optimisation of the care for older people suffering from chronic illnesses and polymorbidity.” | 87.5% (n=14) | 6.2% (n=1) | 6.2% (n=1) |
| “The development of specialist palliative care is less important than improvements in general palliative care to optimise the care for older people suffering from chronic illnesses and polymorbidity.” | 75.0% (n=12) | 12.5% (n=2) | 12.5% (n=2) |
| “This legal entitlement will help to improve domestic care of severely ill and dying people.” | 81.2% (n=13) | 6.2% (n=1) | 12.5% (n=2) |
| “Many employees will not take the unpaid leave for fear of disadvantages in the workplace.” | 62.5% (n=10) | 18.8% (n=3) | 18.8% (n=3) |
