# Supplementary material for: Developing targets for public health initiatives to improve palliative care
Source: BMC Public Health. 2010 Apr 29;10:222. doi: 10.1186/1471-2458-10-222 (PMC2874778; doi:10.1186/1471-2458-10-222)
Supplement: Additional file 4 — Targets, importance and implementation. The table presents the targets for public health initiatives to improve palliative care in Germany, each with its importance and its implementation as assessed in the second Delphi round. [file 1471-2458-10-222-S4.PDF]

| | | | Importance | | | Implementation | | | |
|---|---|---|---|---|---|---|---|---|---|
| **Subject areas** | **No.** | **Targets** | **Median** | **Min** | **Max** | **Median** | **Min** | **Max** | **Number of mentions** |
| palliative care approach | 1 | Sharpening definitions and concepts in palliative care | 2.0 | 0 | 3 | 0.0 | -2 | 1 | 4 |
| | 2 | Clarifying differentiations and overlaps of palliative care with other disciplines | 2.0 | -2 | 3 | 0.0 | -2 | 1 | 3 |
| | 3 | Supporting palliative care as a basic attitude for the care of people in the last phase of life | 3.0 | 1 | 3 | 0.0 | -2 | 2 | 8 |
| patient and family | 4 | Offering support to family members who are caring for someone in the last phase of life | 2.0 | 1 | 3 | -1.0 | -2 | 1 | 5 |
| | 5 | Reducing job-related disadvantages of family members who take care for someone in the last phase of life | 2.0 | 1 | 3 | -2.0 | -3 | 1 | 0 |
| | 6 | Considering the cultural background of people is the last phase of life | 2.0 | 0 | 3 | -1.0 | -2 | 1 | 2 |
| | 7 | Considering the financial situation of people in the last phase of life | 2.0 | -2 | 3 | 0.0 | -2 | 2 | 2 |
| | 8 | Prioritising the quality of life of the people concerned | 3.0 | 2 | 3 | 1.0 | -1 | 2 | 8 |
| health services | 9 | Establishing cooperation among health professions and disciplines | 3.0 | 1 | 3 | -1.0 | -2 | 2 | 7 |
| | 10 | Linking structures of generalist and specialist palliative care services | 2.0 | 1 | 3 | -1.0 | -2 | 1 | 4 |
| | 11 | Coordinating healthcare for people in the last phase of life | 2.0 | 1 | 3 | -1.0 | -2 | 1 | 5 |
| | 12 | Deploying community nurses in the care for people in the last phase of life | 2.0 | 0 | 3 | 0.0 | -3 | 2 | 1 |
| | 13 | Considering regional differences in the determination of requirements for care structures | 2.0 | 1 | 3 | -1.0 | -2 | 1 | 1 |
| | 14 | Planning the demand for specialist palliative care services | 2.0 | 1 | 3 | -1.0 | -3 | 2 | 2 |
| | 15 | Focusing on outpatient palliative care services | 2.0 | 0 | 3 | 0.0 | -2 | 1 | 2 |
| | 16 | Strenghtening primary palliative care | 2.5 | 1 | 3 | -1.0 | -2 | 1 | 4 |
| | 17 | Focusing on the needs of older people in primary palliative care | 2.0 | -1 | 3 | 0.0 | -2 | 1 | 0 |
| Information and qualification | 18 | Informing about contents, targets and opportunities of palliative care on a societal level | 2.0 | 1 | 3 | 0.0 | -3 | 1 | 1 |
| | 19 | Establishing education in palliative care for all professional groups with contact to people in the last phase of life | 3.0 | 1 | 3 | 0.0 | -2 | 1 | 5 |
| | 20 | Standardize qualifications of specialist palliative care professionals | 2.0 | 0 | 3 | -1.0 | -2 | 1 | 1 |
| Reserach | 21 | Reviewing the evidence of palliative care measures | 2.0 | 0 | 3 | -1.0 | -3 | 1 | 6 |
| | 22 | Conducting demand research with regard to specialist palliative care services | 2.5 | 1 | 3 | -1.0 | -3 | 1 | 2 |
| | 23 | Specifying target groups and their need in palliative care | 2.0 | 0 | 3 | -1.0 | -2 | 1 | 3 |
| Financing | 24 | Reducing the financial burden for family members whotake care of people in the last phase of life | 2.0 | 0 | 3 | -1.0 | -2 | 1 | 1 |
| | 25 | Overcoming structural fragmentation of the funding of services in the health system | 2.0 | 1 | 3 | -1.0 | -3 | 0 | 3 |
